# Fetal ECG Signal Extraction from Maternal Abdominal ECG Signals Using Attention R2W-Net

**DOI:** 10.3390/s25030601

**Published:** 2025-01-21

**Authors:** Lin Chen, Shuicai Wu, Zhuhuang Zhou

**Affiliations:** College of Chemistry and Life Sciences, Beijing University of Technology, Beijing 100124, China; chenlin_2023@emails.bjut.edu.cn (L.C.); zhouzh@bjut.edu.cn (Z.Z.)

**Keywords:** fetal electrocardiogram signal extraction, Attention R2W-Net, recurrent residual convolution

## Abstract

Fetal electrocardiogram (FECG) signals directly reflect the electrical activity of the fetal heart, enabling the assessment of fetal cardiac health. To effectively separate and extract FECG signals from maternal abdominal electrocardiogram (ECG) signals, this study proposed a W-shaped parallel network, termed Attention R2W-Net, which consisted of two Attention R2U-Nets. In the encoder and decoder, recurrent residual modules were used to replace feedforward convolutional layers, significantly enhancing feature representation and improving noise suppression. Additionally, attention gates were used to replace skip connections, enabling precise correction of low-resolution features using deep features and further improving model performance. The decoders at both ends of the network were utilized to reconstruct FECG and MECG signals, respectively. The algorithm was validated using simulated and real datasets, achieving F1 scores of 99.17%, 98.03%, and 97.08% on the ADFECGDB, PCDB, and NIFECGDB datasets, respectively, demonstrating superior performance in both subjective visual effects and objective evaluation metrics. Attention R2W-Net’s ability to extract robustly and accurately FECG signals, even in complex noisy environments, make it a reliable tool for FECG extraction. The proposed method’s efficiency and accuracy highlight its potential for widespread clinical application, contributing to improved early diagnosis of fetal cardiac abnormalities.

## 1. Introduction

Non-invasive fetal electrocardiogram (FECG) is an advanced fetal monitoring technology that captures FECG signals by placing a series of electrodes on the abdominal wall of pregnant women. In clinical practice, one can diagnose the health status of the fetus by analyzing the electrocardiogram (ECG) waveform and fetal heart rate. The morphological and temporal changes in specific intervals of FECG signals (such as RR interval, QRS complex, and ST segment) often suggest pathological conditions of the fetus during pregnancy (such as fetal hypoxia and congenital heart disease), including shortened QT and PR intervals of FECG, which are related to metabolic acidosis and congenital heart defects (CHD) caused by hypoxia [1,2]. Therefore, extracting clear FECG signals from maternal abdominal ECG (maECG) signals can help in the timely detection of fetal pathological conditions during pregnancy, thereby preventing neonatal diseases and reducing the risk of fetal death.

The FECG signal embedded in maECG signals is of low amplitude and low signal-to-noise ratio, with significant interference from maternal ECG (MECG) signals [3]. In addition, the QRS waveform duration and shape of the fetus are usually similar to those of the mother, but its amplitude and QRS wave width are relatively small. In some cases, the FECG signals may overlap with the MECG signals, making it challenging to extract clean FECG signals from maECG. To overcome the challenge of extracting clean FECG signals from maECG, researchers have employed various signal processing techniques. The commonly used techniques include the wavelet transform (WT) [4], adaptive noise cancellation (ANC) [5], extended Kalman filtering (EKF) [6], empirical mode decomposition (EMD) [7], and blind source separation (BBS) [8]. The performance of WT-based methods largely depends on the selected wavelet basis function; its computational complexity is high, making it sensitive to noise and interference in the signal. Adaptive filtering requires simultaneous acquisition of maternal chest and abdominal signals, which increases discomfort for pregnant women during measurement, and the performance of adaptive filtering is highly dependent on the quality and characteristics of the input signal. EKF is very sensitive to the estimation of initial state. Due to the special properties of FECG signals, it may be difficult to obtain accurate initial state estimation, which may affect the performance of EKF. The “basis” of EMD lacks a clear analytical expression, and the overall average number of decompositions must be manually set. The EMD-based methods are also prone to mode mixing effects. BBS methods are primarily based on principal component analysis (PCA) and independent component analysis (ICA) [9]. While these methods demonstrate certain advantages in signal separation, they also have notable limitations. As a dimensionality reduction technique, PCA effectively simplifies data and retains most of the information, but it may struggle to preserve the complete morphological features of complex signals, such as FECG signals [10]. ICA, on the other hand, is sensitive to the selection of initial weight vectors. Improper initialization can lead to unstable separation results. When the statistical properties of the signals are not prominent, the algorithm may fail to converge. Additionally, ICA relies on multi-channel data, and its separation performance significantly deteriorates when the input channels are insufficient or when noise interference is severe. In summary, the use of traditional algorithms for extracting FECG signals may suffer from strong dependency on parameters, poor anti-noise robustness, lack of real-time performance, and limited applicability, thereby restricting their widespread adoption in clinical practice.

With the development of deep learning technologies, researchers have explored the application of deep neural networks in the extraction of FECG signals. Deep learning architectures such as convolutional neural networks (CNNs) and recurrent neural networks (RNNs) have been used to learn about useful features from raw signals [11,12]. Compared to traditional algorithms, deep learning can directly extract FECG signals without removing MECG signals, avoiding the errors and signal loss that may arise when using traditional methods. Although deep learning methods have shown advantages in FECG signal extraction, there are still some limitations. For example, when MECG signals and FECG signals overlap significantly or noise levels are high, the accuracy of existing methods decreases. Additionally, some methods fail to effectively preserve the critical waveform features of FECG signals, such as the P-wave and T-wave, which are essential for clinical diagnosis. This paper aims to address these issues by proposing a novel deep learning framework to enhance the robustness and accuracy of signal extraction.

To address the above issues, this paper proposed a W-shaped network based on two one-dimensional (1D) Attention R2U-Nets, named Attention R2W-Net, for FECG signal extraction. Compared to the traditional W-Net architecture [13], the Attention R2W-Net achieves multi-task feature separation through parallel processing rather than the sequential feature optimization used in traditional W-Net. This design demonstrated significant advantages in separating MECG and FECG signals. The proposed model employed an end-to-end approach to extract clear FECG signals from single-channel maECG signals. The input signal underwent feature extraction and down-sampling through a recurrent residual convolution (RRC) block in the encoder. Subsequently, in the decoder, the signal was progressively restored through recursive up-sampling with attention gates (AGs) replacing skip connections to integrate features at different resolutions. Finally, high-quality FECG signals were separated through signal subtraction and the application of the tanh activation function.

The main contributions of this paper are as follows:

(1) An end-to-end deep learning algorithm for single-channel extraction of FECG signals was developed.
(2) The Attention R2W-Net effectively extracts clean FECG signals from maECG while preserving the morphological characteristics of the FECG signals, enabling the detection of P-wave and T-wave information.
(3) The use of RRC blocks in both the encoder and decoder enhances feature extraction depth and signal representation capability, while residual connections retain original feature information, assisting the network in better signal separation and reconstruction.

## 2. Related Work

In recent years, with the growing demand for fetal health monitoring, researchers have proposed various deep learning algorithms for extracting FECG signals from maternal maECG signals.

Shokouhmand et al. [14] proposed a Dual-Path Source Separation (DPSS) architecture for non-invasively separating FECG and MECG signals from maECG signals. The DPSS used a Dual-Path Long Short-Term Memory (DP-LSTM) network to denoise the maECG recordings and separate the FECG and MECG signals by extracting masks. The method showed high accuracy in detecting fetal QRS waves. However, the DP-LSTM network architecture may lead to high computational complexity, which could be a limitation for real-time monitoring and low-power device applications. Additionally, the method relies on the accuracy of the masks, and noise interference or abnormal signals may affect mask generation, thereby reducing the separation performance.

Ghonchi et al. [15] proposed an autoencoder model based on dual attention for FECG signal extraction. The model integrates dual attention modules (including squeeze-and-excitation modules and channel attention modules) with bidirectional Long Short-Term Memory (LSTM) layers, effectively capturing and extracting feature patterns related to the FECG signal without prior MECG information. However, when the signal-to-noise ratio is low or the fetal QRS waves are too weak, the model’s extraction performance significantly deteriorates. Due to the introduction of the dual attention modules and bidirectional LSTM layers, the model has higher complexity and requires further optimization or compression to meet real-time application requirements.

Wei et al. [16] proposed a novel method for extracting FECG signals using Short Time Fourier Transform (STFT) and a Generative Adversarial Network (GAN). First, STFT was applied to transform the maECG signal from the 1D time domain to the two-dimensional (2D) time-frequency domain. Next, the GAN model was used to estimate the 1D-STFT coefficients of the FECG in the time-frequency domain. Finally, after applying the inverse STFT, the FECG was reconstructed in the time domain. The experimental results on two databases demonstrated the effectiveness of their method. However, converting ECG signals into time-frequency maps increases computational complexity, which reduces network efficiency.

Basak et al. [17] proposed a method based on a 1D-CycleGAN model to extract FECG signals from maECG while preserving the morphology of the MECG signals. Their model extracted FECG from four-channel maECG and incorporated a novel loss function, a weighted loss combining temporal, spectral, and power losses. This ensured the accurate reconstruction of FECG signals while maintaining resilience to human errors, thereby improving the overall performance of their algorithm. However, the computational load of the network is relatively high, and the quality of the extracted FECG signals can be further improved if the maECG signal quality is higher. Subsequent improvements can be made to the preprocessing stage to enhance the quality of FECG extraction, or to eliminate maECG signals with low quality by filtering the original maECG signals.

Lee et al. [18] employed a W-Net end-to-end deep learning model to separate maternal and FECG signals. The model consisted of two U-Net networks, with channel sizes of 16, 32, 64, 128, and 256 at each layer, enabling the extraction of multi-scale features in the encoding phase. The model was effective in extracting FECG signals from both simulated and real datasets. However, the extracted FECG signals still contain noise, and the morphological characteristics of the waveforms were not fully captured, making it difficult to identify the P-wave and T-wave shapes. To address these issues, future improvements could involve training the model with real datasets rather than relying solely on simulated data.

## 3. Methodology

### 3.1. Overall Framework

**Figure 1:**
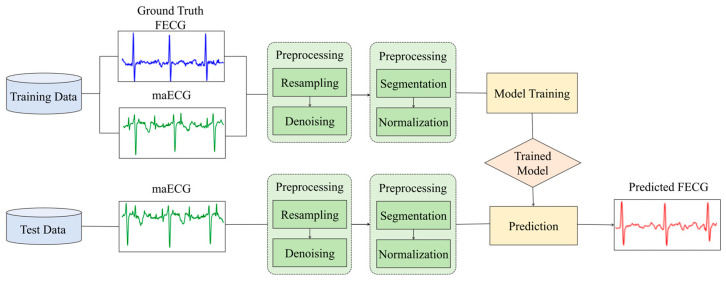
Block diagram of the proposed method for extracting FECG from maECG.

The overall framework of the proposed model is shown in Figure 1. maECG signals were collected using a four-channel ECG acquisition device placed on the maternal abdomen. To suppress noise, the signals first underwent preprocessing steps, including resampling, denoising, segmentation, and normalization. The preprocessed signals were then used to train the model. After training was completed, the model was tested using data from different subjects to obtain the predicted FECG signals. Finally, the optimal model was determined based on two evaluation metrics: the quality of the extracted FECG signals and fetal QRS (FQRS) detection.

### 3.2. Dataset Description

#### 3.2.1. Simulation Data

The PhysioNet Fetal ECG Synthetic Database (FECGSYN) dataset was used to train the proposed model. The dataset can be accessed at https://archive.physionet.org/physiobank/database/fecgsyndb/ (accessed on 1 September 2022). FECGSYNDB is a database that simulates adults and non-invasive FECG signals. It uses the FECGSYN synthesis generator to create simulated data, consisting of 32 abdominal signals and 2 chest signals. The dataset included data from 10 pregnant subjects, and for each subject, 7 different physiological events were simulated. (i) Baseline: Abdominal mixture (no noise or events). (ii) C0: A baseline state with no significant physiological events affecting the signal, but with the presence of noise. (iii) C1: Fetal activity within the uterus, causing irregular fluctuations in the ECG, accompanied by noise. (iv) C2: Acceleration or deceleration of maternal heart rate (MHR) or fetal heart rate (FHR), often associated with the fetus’s response to external stimuli or hypoxia, accompanied by noise. (v) C3: Uterine contractions of the mother, potentially causing fluctuations in the ECG signal, accompanied by noise. (vi) C4: Ectopic beats from both the fetus and the mother, potentially interfering with the ECG signals, accompanied by noise. (vii) C5: Twin pregnancies, where the abdominal electrodes record ECG signals from two fetuses, increasing the complexity of the signal, accompanied by noise. For each physiological event, five levels of additive noise (0, 3, 6, 9, and 12 dB) were applied. Each scenario was simulated five times, resulting in a total of 1750 synthetic signals, with each signal sampled for 5 min at a frequency of 250 Hz.

The data from the 10th subject was used as the test dataset, while the remaining subjects were used as the training dataset to evaluate model performance. Training was conducted using maECG signals from channels 11, 19, 22, and 25.

#### 3.2.2. Real Data

In this study, the model was fine-tuned using a subset of the Abdominal and Direct Fetal Electrocardiogram Database (ADFECGDB), while the remaining data were used for model testing. The dataset can be accessed at https://physionet.org/content/adfecgdb/1.0.0/ (accessed on 1 September 2022). The ADFECGDB contains FECG data from five women in different stages of pregnancy (38–41 weeks). The dataset includes five data channels: four abdominal channels and one direct fetal scalp channel. Each recording lasts 5 min with a sampling frequency of 1 kHz.

To further validate the accuracy of the proposed algorithm, this study also utilized set A of the 2013 PhysioNet/Computing in Cardiology Challenge database (PCDB). The dataset can be accessed at https://physionet.org/content/challenge-2013/1.0.0/ (accessed on 1 September 2022). This dataset comprises 75 non-invasive abdominal mixed ECG recordings, with each record containing four channels of mixed signals obtained from the maternal abdomen. The signals were sampled at a frequency of 1000 Hz for a duration of 60 s per record. Each record has been manually annotated by ECG experts to identify the fetal R-peaks.

This study also utilized the Non-Invasive Fetal ECG Database (NIFECGDB) for validation, which can be accessed at https://physionet.org/content/nifecgdb/1.0.0/ (accessed on 1 September 2022). This dataset consists of 55 multi-channel recordings, including 3–4 abdominal signals and 2 thoracic signals. The sampling frequency is 1 kHz, and fetal heartbeat annotations are publicly available. For comparison with the state-of-the-art results [15], 14 subsets were selected, including 154, 192, 244, 274, 290, 323, 368, 444, 597, 733, 746, 811, 826, and 906.

### 3.3. The Proposed Method

#### 3.3.1. Preprocessing

**Figure 2:**
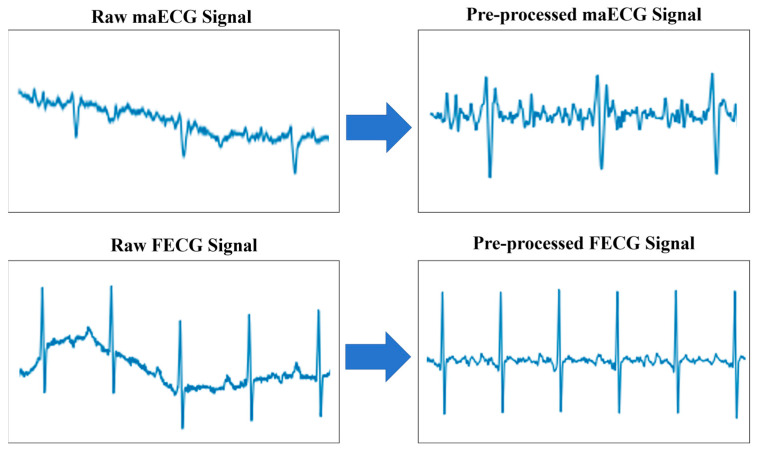
Comparison of pre-processing of maECG and FECG signals.

The maECG signals collected contained noise such as pulse artifacts, baseline drift, and powerline interference [19]. Therefore, preprocessing the maECG signals was crucial. Since the frequency range of the QRS peaks in FECG signals was between 10 and 15 Hz [20], with a minimum bandwidth of 100 Hz [21], a 100 Hz low-pass filter and a 3 Hz high-pass filter were used to remove high-frequency noise and low-frequency noise, respectively. As shown in Figure 2, after the filtering, major noise components such as pulse artifacts, baseline drift, and power line interference were effectively removed, significantly improving the signal quality and laying a solid foundation for subsequent signal analysis and processing. Given the differing sampling frequencies across databases, the raw signals were resampled to 250 Hz. Subsequently, the maECG signals were then divided into segments of 1024 sample points, with an overlap of 24 sample points at both ends to ensure signal continuity. Finally, the maECG signals were normalized using Z-score [22].

#### 3.3.2. Model Architecture

##### Attention R2W-Net Architecture 

**Figure 3:**
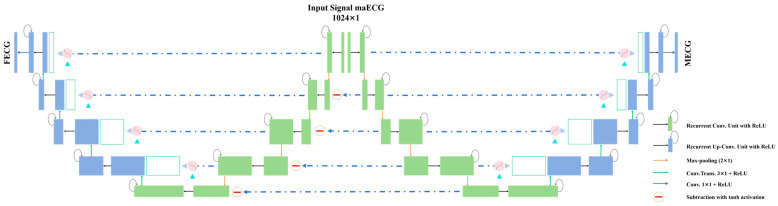
The FECG signal extraction model based on Attention R2W-Net.

The proposed Attention R2W-Net framework is illustrated in Figure 3. The network was composed of two Attention R2U-Nets, forming a W-shaped architecture. The Attention R2U-Net on the right was used for MECG extraction, while the one on the left was used for FECG extraction. Both Attention R2U-Nets on the left and right sides contained a five-layer convolutional structure, which comprised two main units: the encoding unit (in green) and the decoding unit (in blue). In the encoder on the left, features of the FECG were extracted by subtracting the features of the MECG extracted by the encoder on the right. Finally, the FECG and MECG signals were reconstructed in the decoders at both ends. This subtraction operation employed the tanh activation function to enhance the effectiveness of feature extraction.

##### Encoder and Decoder

In both the decoder and encoder, the most important structure was the RRC block. Suppose that in the l-th layer of the RRC block, the input signal is $x_{l}$. Let $\left(i,j\right)$ represent the spatial location in the feature map, and the signal is located in the k-th feature map channel. The output $Y_{ijk}^{l}\left(t\right)$ at time step t can be expressed as

(1) $$Y_{ijk}^{l}\left(t\right)={\left(w_{k}^{f}\right)}^{T}x_{l}^{f\left(i,j\right)}\left(t\right)+{\left(w_{k}^{r}\right)}^{T}x_{l}^{r\left(i,j\right)}\left(t-1\right)+b_{k}$$

where $x_{l}^{f\left(i,j\right)}\left(t\right)$ and $x_{l}^{r\left(i,j\right)}\left(t-1\right)$ represent the outputs of the standard convolution and recurrent convolution at the l-th layer, respectively. $w_{k}^{f}$ and $w_{k}^{r}$ denote the weights of the standard convolution layer and recurrent convolution layer, respectively. $b_{k}$ represents the bias term.

The output is processed using the rectified linear unit (ReLU) activation function:

(2) $$F\left(x_{l},w_{l}\right)=f\left(Y_{ijk}^{l}\left(t\right)\right)=\max\left(0,Y_{ijk}^{l}\left(t\right)\right)$$

The output of the R2CNN block is given by

(3) $$x_{l+1}=x_{l}+F\left(x_{l},w_{l}\right)$$

where $x_{l}$ represents the input of the RRC block. $x_{l+1}$ refers to the output of the downsampling layer in the encoding path or the output of the upsampling layer in the decoding path.

**Figure 4:**
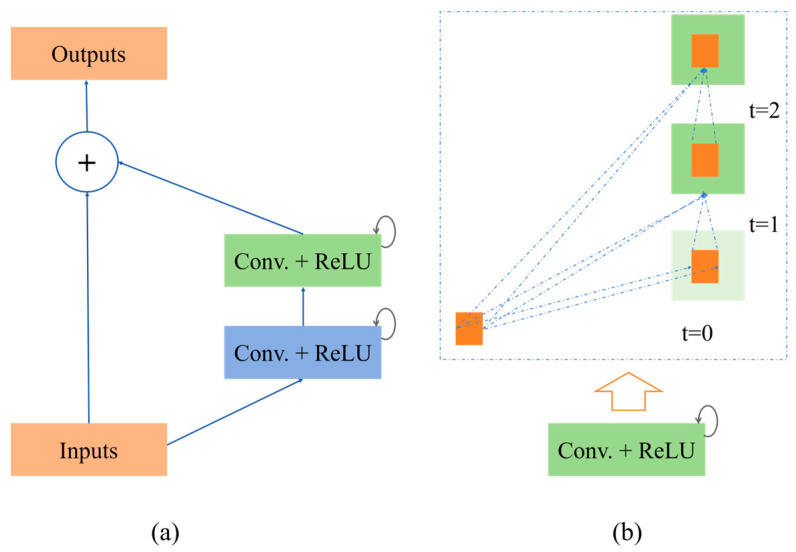
(**a**) The recurrent residual convolutional unit and (**b**) the unfolded version of the recurrent convolutional unit.

The conceptual diagram of the RRC block is shown in Figure 4. In this paper, T = 2, meaning that one general convolutional layer and two recurrent layers were used in the convolutional unit. During time series processing, the convolutional and recurrent layers alternate operations over two time steps. The choice of convolutional kernel sizes affected the network’s ability to capture signal features and preserve details. Smaller kernels were suitable for extracting local features, while larger kernels could capture more contextual information [23]. Therefore, for the network that extracted MECG and FECG, the R2CNN modules consisted of convolutions with kernel sizes of 5 and 3, respectively, with added recurrent connections and residual connections in each convolutional layer. This design captured temporal features in the data and enhanced contextual information patterns, contributing to construct a deeper model [24].

The encoder of the network at both ends consisted of five RRC blocks and four max-pooling layers. The channel sizes of each layer were 64, 128, 256, 512, 256, and 1024, respectively, enabling the extraction of features at multiple scales in the encoding process. The RRC blocks captured features at different depths and temporal patterns in the sequential data, while the pooling layers reduced the spatial dimensions of the feature maps. Specifically, the input data first passed through the first RRC module and a max-pooling layer, reducing the size of the feature map while extracting deep features. This process was repeated through the second, third, fourth, and fifth RRC modules and their corresponding pooling layers, progressively extracting deeper-level features. Ultimately, the input data were encoded into a compact high-level feature representation.

**Figure 5:**
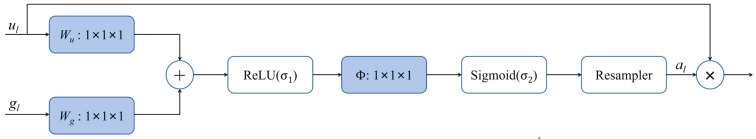
Structure diagram of the AG module.

The decoder was symmetric to the encoder and was responsible for progressively restoring the compact high-level features generated by the encoder back to the size of the input data. The decoder consists of four upsampling layers and five RRC blocks. The decoder restored features through upsampling and adjusted the encoder’s output features with the help of AG modules. It concatenated the encoder’s feature maps at the corresponding resolutions with those of the decoder. This module generated a gating signal to control the importance of features at different positions. The final convolutional layer was used to generate the output with the required number of channels. As shown in Figure 5, in the AG module, the feature output of the encoder $u_{l}$ and the gating signal $g_{l}$ were used as inputs to the AG, representing the feature map at the current decoding stage and the gating signal used to guide attention distribution, respectively. The intermediate result was first calculated through the following additive attention formula:

(4) $$Q_{L}=\Phi \left(\sigma_{1}\left(W_{u}u_{l}+W_{g}g_{l}+b_{g}\right)\right)+b_{\Phi }$$

where $W_{u}$ and $W_{g}$ are the weights of the features $u_{l}$ and $g_{l}$, respectively; $b_{g}$ and $b_{\Phi }$ are the bias terms; $\sigma_{1}$ is the ReLU activation function; and Φ represents the output function of the linear combination.

Next, the final attention coefficient $\alpha_{l}$ is generated through the Sigmoid activation function $\sigma_{2}$:

(5) $$\alpha_{l}=\sigma_{2}\left(Q_{L}\right)$$

where $\alpha_{l}$ represents the attention weight for each sample point, used to emphasize key regions (such as the feature areas of the signal) while suppressing irrelevant regions.

In FECG signal extraction, AGs can effectively identify and highlight the feature areas of the fetal signal, while reducing the interference from maternal signals and background noise, thereby improving the model’s accuracy and robustness in extracting the target signal.

#### 3.3.3. Training Parameters

In this study, Python 3.8 was used as the programming language, and the PyTorch and MONAI Python packages were employed to train the model. The workstation was equipped with two Intel(R) Xeon(R) Gold 6132 CPUs @ 2.60 GHz/2.59 GHz, an NVIDIA TITAN RTX 24 GB GPU, and 128 GB of RAM. The mean absolute error (MAE) was selected as the loss function due to its simplicity, robustness to outliers, linear loss characteristics, stable convergence performance, and flexibility across various scenarios. To ensure stable signal amplitudes, zero-padding was applied in all convolution and deconvolution operations, with a stride set to 1. The activation function for each layer was chosen to be the Leaky ReLU, providing the network with greater flexibility and nonlinear expressiveness. We used Adam as the optimizer for model training, which combined the advantages of Momentum and RMSprop. It dynamically adjusted the learning rate for each parameter during training, accelerating convergence and improving training efficiency. The initial learning rate was set to 0.0001, which ensured stable convergence of the model without skipping the optimal solution too quickly, and helped avoid issues such as gradient explosion or gradient disappearance. The batch size was set to 32, and the number of epochs was set to 30. This configuration balanced training efficiency and convergence stability, with overfitting prevention, allowing the model to better learn the data features and generalize effectively.

### 3.4. Performance Evaluation Methods

To evaluate the performance of the FECG signal extraction algorithm, two different evaluation methods can be employed for comparison: objective evaluation methods and subjective evaluation methods.

#### 3.4.1. Objective Evaluation Methods

##### Evaluation of FECG Signal Extraction Quality

The quality of the extracted FECG signal was evaluated using mean squared error MSE, mean absolute error MAE, and signal-to-noise ratio SNR:

(6) $$\mathrm{MSE}=\frac{1}{n}\sum_{i=1}^{n}{\left(y_{i}-{\hat{y}}_{i}\right)}^{2}$$

(7) $$\mathrm{MAE}=\frac{1}{n}\sum_{i=1}^{n}\left|y_{i}-{\hat{y}}_{i}\right|$$

(8) $$\mathrm{SNR}=10\times \log_{10}\left(\frac{\sum_{i=1}^{n}{y_{i}}^{2}}{\sum_{i=1}^{n}{\left(y_{i}-{\hat{y}}_{i}\right)}^{2}}\right)$$

where $y_{i}$ represents the true FECG signal amplitude, ${\hat{y}}_{i}$ represents the extracted FECG signal amplitude, and *n* is the total number of samples in the signal. Both MSE and MAE were used to measure the discrepancy between the model’s predictions and the actual values. Smaller values of MSE and MAE indicated better model performance, showing that the extracted FECG signal was closer to the true signal. SNR was used to assess the clarity of the signal; thus, a higher SNR value signified less noise in the signal and better quality of the extracted FECG signal [25].

##### Evaluation of FECG R-Peak Detection

The equations for calculating sensitivity SE, positive predictive value PPV, and F1 score are as follows:

(9) $$\mathrm{Se}=\frac{\mathrm{TP}}{\mathrm{TP}+\mathrm{FN}}\times 100\%$$

(10) $$\mathrm{PPV}=\frac{\mathrm{TP}}{\mathrm{TP}+\mathrm{FP}}\times 100\%$$

(11) $$F1=\frac{\mathrm{TP}}{\mathrm{TP}+\mathrm{FP}+\mathrm{FN}}\times 100\%$$

In the evaluation of R-peak detection in FECG signals, the correctly identified R-peaks were referred to as true positives (TPs), indicating the positions where the detection method accurately marked the R-peaks. The incorrectly marked R-peak positions were called false positives (FPs), representing R-peaks that did not actually exist but were falsely detected. Additionally, the actual R-peaks that were not detected were categorized as false negatives (FNs), representing those R-peaks that should have been detected but were missed. Higher values of sensitivity (SE), positive predictive value (PPV), and F1 score indicated better performance of the FECG signal extraction method.

#### 3.4.2. Subjective Evaluation Metrics

By observing the clarity and completeness of the extracted FECG signals, we assessed whether the algorithm effectively reduced the interference from MECG signals and noise. This intuitive approach enabled the comparison of performance across different algorithms.

## 4. Results

### 4.1. FECG Extraction Results

The FECG signals extraction algorithm proposed in this paper was experimentally validated using test datasets. The test datasets consisted of data from the 10th subject of the FECGSYN dataset, test data from the ADFECGDB database, and data from the PhysioNet 2013 Challenge database.

The effectiveness of the proposed model was validated on the FECGSYN dataset. As shown in Table 1, the proposed algorithm achieved high-quality FECG signal extraction under various noise conditions. Even in more complex noise environments, such as uterine contractions (C3) and ectopic beats (C4), the algorithm still demonstrated strong performance. This further confirmed the generalizability and robustness of the proposed algorithm in handling complex noise.

The performance of the proposed algorithm for FECG signal extraction was evaluated on the ADFECGDB database. As shown in Table 2, the Attention R2W-Net achieved an MSE of 0.025, an MAE of 0.013, and an SNR of 8.03 dB.

**Figure 6:**
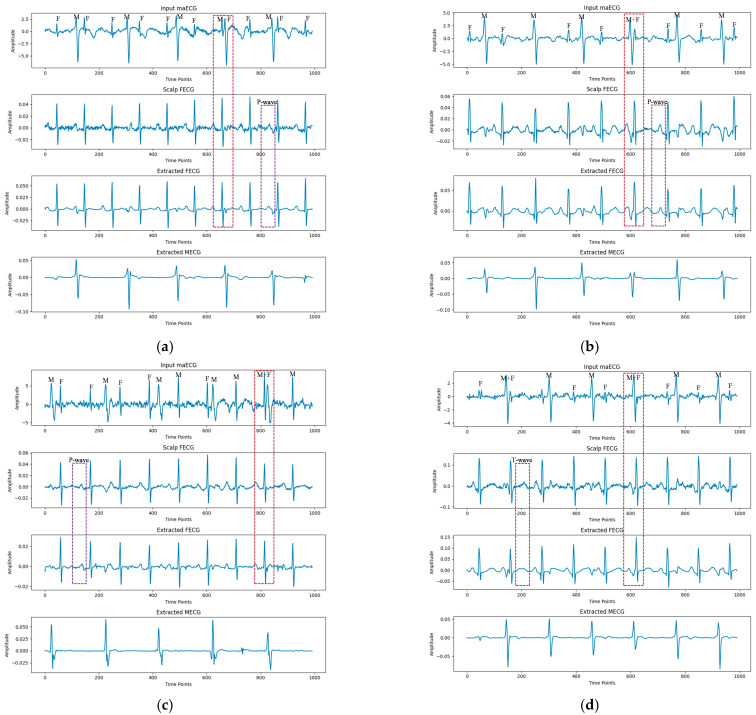
Examples of FECG extraction on the ADFECGDB database by the proposed method. (**a**–**d**) represent channels I, II, III, and IV, respectively. The positions of the FECG signal, MECG signal, and MECG signal overlapping with the FECG signal in the maECG signal are indicated by ‘F’, ‘M’, and ‘F + M’, respectively.

As shown in Figure 6, data from the ADFECGDB database were selected for an intuitive demonstration of the algorithm’s extraction performance. The FECG signals extracted using the proposed algorithm were compared with the direct fetal scalp signals from the database. The extracted FECG signals were smoother and clearer, and the QRS waves were accurately extracted. The algorithm addressed the issue of difficult FECG signal extraction in areas where MECG components partially overlapped with FECG components (red dashed box in Figure 6a,c), and where they completely overlapped (red dashed box in Figure 6b,d). The proposed algorithm successfully extracted MECG signals while separating FECG signals. Additionally, the algorithm effectively reconstructed P and T waves in challenging cases, as indicated by the purple rectangular box. The algorithm also succeeded in extracting FECG signals from different channels; for the four-channel data in the ADFECGDB database, it accurately extracted FECG signals from each channel, demonstrating its capability to extract FECG signals using a single channel.

**Figure 7:**
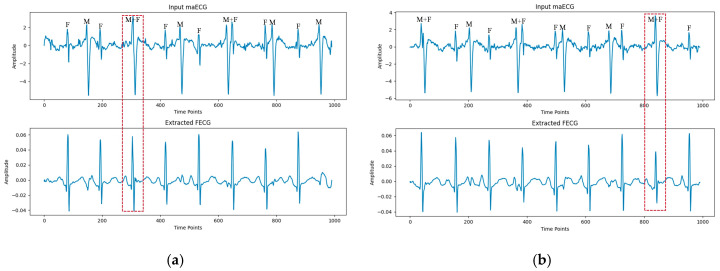
Examples of FECG extraction on the PCDB database by the proposed method. (**a**,**b**) represent channels I and II of data a03, respectively. The positions of the FECG signal, MECG signal, and MECG signal overlapping with the FECG signal in the maECG signal are indicated by ‘F’, ‘M’, and ‘F + M’, respectively.

Similarly, as shown in Figure 7, in the PhysioNet 2013 Challenge database, the proposed algorithm effectively suppressed residual MECG components while extracting clear FECG waveforms. This resolved the issue of missing FECG waveforms when maternal and fetal signals overlapped (red dashed box in Figure 7). Therefore, the model can extract clear FECG signals from complex maECG signals while preserving the morphological information of the FECG signals.

To further validate the proposed algorithm, our model was compared with existing deep learning models across multiple databases. As shown in Table 3, compared with state-of-the-art deep learning models, our model performed excellently, with relatively low MSE and MAE. Our model can better capture key features in signals and improve the quality of FECG signal extraction. Additionally, to assess the significance of the performance differences, statistical significance tests were performed on the performance metrics of each model across multiple databases. The *T*-test, a commonly used method in statistics for assessing the significance of experimental data, was applied. As shown in Table 4, all *p*-values were less than 0.05, indicating that at a 5% significance level, the performance differences between our model and other existing models were significant. This result further supports the superiority of our model in terms of performance.

### 4.2. FQRS Detection Results

When the time difference between the R peaks detected by the proposed algorithm and the actual R peaks was within 31.25 ms, the FQRS complex was considered to be correctly extracted. The Pan–Tompkins algorithm was used for FQRS complex detection on the FECG signals extracted using the Attention R2W-Net.

Table 5 presents the FQRS complex detection results on the ADFECGDB database, with the average SE, PPV, and F1 score being 99.24%, 99.11%, and 99.17%, respectively. The Attention R2W-Net model demonstrated exceptional performance, successfully extracting FQRS complexes. It maintained efficient detection capabilities under complex and challenging conditions, further proving its reliability and effectiveness in practical applications.

The proposed FECG signal extraction algorithm was compared with other advanced deep learning algorithms on the FECGSYNDB database, ADFECGDB database, PhysioNet 2013 Challenge database, and NIFECGDB database. The results are presented in Table 6.

From Table 6, it can be seen that our algorithm achieved superior performance in most cases. Although Mohebbian et al. proposed an attention-based CycleGAN [22] network that achieved an F1 score of 99.70 on the ADFECGDB dataset, the CycleGAN had high computational complexity, making it challenging to use in real time. Additionally, their network failed to detect the P waves and T waves in some challenging cases. Almadani et al. employed the W-NETR [26] network to extract FECG signals, achieving satisfying FQRS detection performance. However, the high computational complexity, dependency on the quality of the input signals, and insufficient robustness to certain types of noise were factors that need to be considered in practical applications.

### 4.3. Ablation Experiments

To comprehensively evaluate the performance and advantages of the proposed model, we conducted ablation experiments to verify the impact of different design choices on the model’s performance.

**Figure 8:**
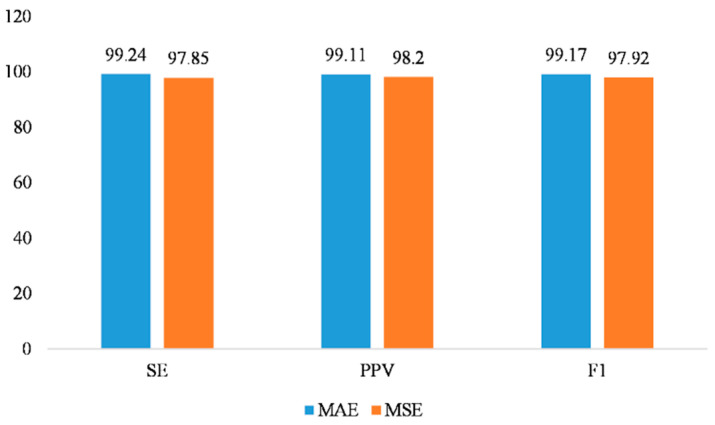
The impact of loss functions on model performance.

For the choice of the loss function, we compared the performance of mean squared error MSE and mean absolute error MAE during the model training. Using the ADFECGDB dataset for validation, the experimental results demonstrated that the model’s performance in FECG signal extraction was superior when using MAE as the loss function compared to MSE, as shown in Figure 8.

MSE tended to amplify the impact of large error values, making the model overly sensitive to outliers. The maECG signals often contained noise, and MSE could lead to unstable learning outcomes for the model due to noise interference, especially in cases of significant noise or irregular waveforms. In contrast, MAE was less sensitive to large errors and could more smoothly handle anomalous data, resulting in a better fit for the overall signal. This was particularly effective in dealing with noise in maECG signals. Therefore, we chose MAE as the loss function, which effectively enhanced the accuracy of FECG signal extraction.

**Figure 9:**
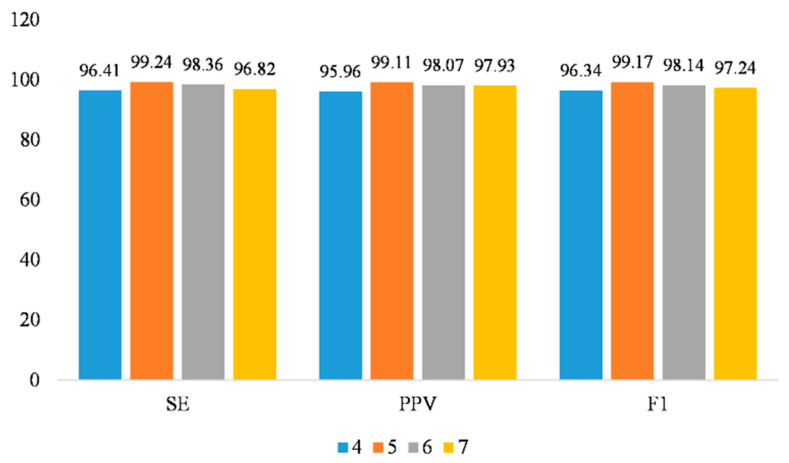
The impact of the number of convolutional layers on model performance.

Additionally, we explored the impact of the number of convolutional layers on the model’s performance. By comparing experimental results with different numbers of convolutional layers, we found that the model achieved the best signal extraction performance when the number of convolutional layers was set to 5, as shown in Figure 9. Specifically, a smaller number of convolutional layers (such as 4) might be insufficient to capture the complex signal characteristics, leading to less precise extraction results. Especially when dealing with FECG signals, which were relatively complex and highly variable, a limited number of convolutional layers might fail to effectively extract sufficient spatial and temporal features, resulting in imprecise signal extraction. Conversely, an excessive number of convolutional layers (such as 6 or more) could introduce overfitting issues, making the model more sensitive to noise and thereby reducing the effectiveness of signal extraction. Experimental validation demonstrated that 5 convolutional layers produced the optimal balance between complexity and the ability to capture signal characteristics, enhancing the accuracy of FECG signal extraction while mitigating overfitting.

**Figure 10:**
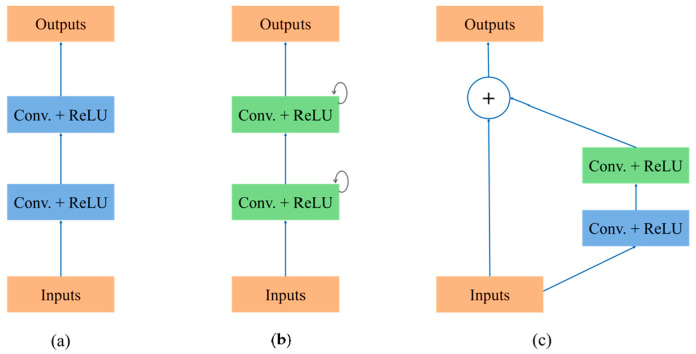
Structural diagrams of the forward convolution block (**a**), the recurrent convolution block (**b**), and the residual convolution block (**c**).

To evaluate the impact of each module in the Attention R2W-Net on network performance, we conducted ablation experiments by replacing the original network’s RRC module with forward convolution blocks, residual convolution blocks, and recurrent convolution blocks, respectively, to compare the performance of different structures in the FECG signal extraction task. Specifically, forward convolution block (Figure 10a) adopted the most basic forward convolution unit, consisting of two standard convolution layers followed by the ReLU activation function. Recurrent convolution block (Figure 10b) was constructed by stacking recurrent convolution units. Residual convolution block (Figure 10c) introduced residual connections based on the forward convolution. The proposed RRC (Figure 4a) combined residual connections and recurrent convolutions.

As shown in Table 7, under the same training configuration, the experimental results show that the forward convolution block only had basic spatial feature extraction capabilities, resulting in relatively lower signal extraction performance. The residual convolution block, by introducing residual connections, effectively alleviated the gradient vanishing problem and improved the network’s convergence speed and extraction accuracy. The recurrent convolution block captured temporal features through temporal modeling, demonstrating its advantage in handling sequential signals. In contrast, the recurrent residual convolution block combined the strengths of residual connections and recurrent convolutions, mitigating gradient vanishing issues while enhancing temporal feature modeling, ultimately achieving the best FECG signal extraction accuracy.

## 5. Discussion

This paper proposes a W-shaped network structure based on Attention R2U-Net for the task of FECG signal extraction. The experimental results show that the network can effectively extract FECG signals from maECG signals with high accuracy and robustness. By introducing RRC units and an attention mechanism, the proposed network is able to extract and fuse features at different resolutions, enhancing its ability to capture key features. This is particularly useful in noisy environments, where the network is still able to accurately separate FECG signals. Compared to traditional FECG extraction methods, the proposed approach demonstrates significant performance improvement.

**Figure 11:**
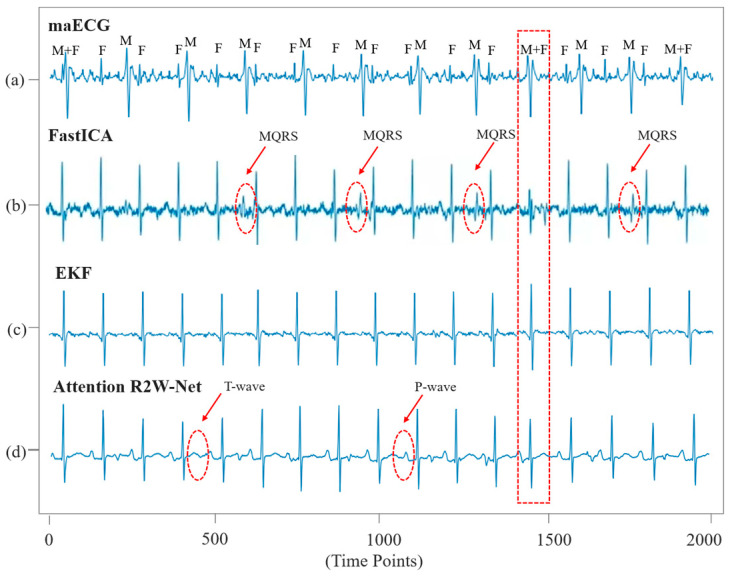
Comparison of the effectiveness of different algorithms in extracting FECG signals, (**a**) maternal abdominal ECG signal; (**b**) FECG extracted by FastICA; (**c**) FECG extracted by EKF; (**d**) FECG extracted by Attention R2W-Net.

Traditional algorithms for FECG extraction typically first remove the MECG signal before extracting the FECG. In contrast, the proposed network can simultaneously separate MECG and FECG signals. FECG signals are typically similar to MECG signals, and traditional FECG extraction algorithms, such as FastICA and EKF, perform poorly when dealing with complex nonlinear signals, making it challenging to accurately separate FECG. Particularly in the presence of strong noise and signal overlap, these algorithms may result in the loss or distortion of FECG waveforms, thereby affecting the diagnosis of the ECG waveform. For example, the FECG signals extracted using FastICA still contain residual MECG interference (Figure 11b) and perform poorly when maternal and fetal signals overlap. While the EKF algorithm achieves relatively better extraction quality, it fails to preserve the characteristics of the P-wave and T-wave in the FECG (Figure 11c). Our network effectively addresses the issue of waveform loss during FECG extraction while preserving the morphological characteristics of the FECG signal. This is primarily due to the effective removal of maternal features in the encoder and the introduction of RRC units, which capture the temporal dependencies in the signal. As a result, the network is able to better capture both local and global features of the signals, thereby effectively retaining the waveform morphology of the FECG signals.

To analyze the complexity of our model, we compared it with four mainstream deep learning models, all tested in the same environment. The experimental results showed that our model required only 0.18 s to extract the FECG signal from a 4 s maECG signal, outperforming CycleGAN [28], PA^2^NET [27], DPSS [14], and W-NETR [30] which required 0.20 s, 0.28 s, 0.46 s and 0.34 s, respectively. This result indicated that Attention R2W-Net had a lower computational complexity, making it suitable for real-time fetal monitoring systems.

The Attention R2W-Net model showed significant performance in FECG signal extraction, but there are still limitations. With the introduction of more high-quality and diverse real datasets, the model’s generalization ability can be further enhanced. Additionally, exploring the combination of more advanced attention mechanisms and convolutional structures can help optimize the model’s complexity and computational efficiency, making it suitable for real-time processing and low-power device applications. In summary, the proposed model has broad application prospects in the field of FECG signal extraction and is expected to provide more precise and reliable solutions for fetal health monitoring.

## 6. Conclusions

To extract clean and high-quality FECG signals from maECG, this study designed an Attention R2W-Net model for end-to-end FECG extraction. This model combines the advantages of attention mechanisms, RRC units, and multi-scale convolutions, effectively enhancing the precision of target signal extraction, suppressing noise, and improving feature representation capability. The algorithm was validated on multiple datasets, demonstrating its ability in extracting high-quality FECG signals while effectively preserving the morphological characteristics of the FECG. Additionally, it achieved a high F1 score in FQRS detection. The model’s robustness and anti-noise capability make it more adaptable in real-world environments, providing clear FECG signals even in cases of high noise levels in maECG, thereby significantly improving the quality and reliability of fetal monitoring.

## Figures and Tables

**Table 1:** Evaluation metrics of FECG signal extraction on FECGSYN by the proposed method.

	C0	C1	C2	C3	C4	C5	Total
MSE	0.079	0.083	0.081	0.122	0.114	0.097	0.096 ± 0.016
MAE	0.075	0.077	0.082	0.093	0.085	0.079	0.082 ± 0.006

**Table 2:** Evaluation metrics of FECG signal extraction on ADFECGDB by the proposed method.

Data	MSE	MAE	qSNR
R01	0.019	0.009	9.08
R04	0.024	0.013	7.17
R07	0.031	0.017	7.94
R08	0.025	0.010	8.41
R10	0.027	0.014	7.53
MEAN ± STD	0.025 ± 0.004	0.013 ± 0.003	8.03 ± 0.67

**Table 3:** Comparisons of different methods for FECG extraction on FECGSYNDB and ADFECGDB datasets.

Method	Database	MSE	MAE
RCED-Net [26]	FECGSYNDB	0.171 ± 0.015	0.128 ± 0.012
	ADFECGDB	0.061 ± 0.006	0.019 ± 0.005
PA^2^NET [27]	FECGSYNDB	0.249 ± 0.039	0.172 ± 0.027
	ADFECGDB	0.146 ± 0.014	0.098 ± 0.007
CycleGAN [28]	FECGSYNDB	0.109 ± 0.011	0.092 ± 0.009
	ADFECGDB	0.042 ± 0.008	0.011 ± 0.004
LinkNet++ [29]	ADFECGDB	0.089 ± 0.002	0.068 ± 0.011
AEDL [15]	ADFECGDB	0.059 ± 0.002	0.018 ± 0.003
The proposed Attention R2W-Net	FECGSYNDB	0.096 ± 0.016	0.082 ± 0.006
	ADFECGDB	0.025 ± 0.004	0.013 ± 0.003

**Table 4:** *T*-test Comparison of different methods for FECG extraction on FECGSYNDB and ADFECGDB datasets.

Method	Database	P_MSE	P_MAE
RCED-Net [26]	FECGSYNDB	8.066 × 10^−6^	5.040 × 10^−5^
	ADFECGDB	1.066 × 10^−5^	5.743 × 10^−2^
PA^2^NET [27]	FECGSYNDB	6.333 × 10^−5^	3.212 × 10^−4^
	ADFECGDB	1.531 × 10^−5^	8.343 × 10^−7^
CycleGAN [28]	FECGSYNDB	1.359 × 10^−2^	4.974 × 10^−2^
	ADFECGDB	5.626 × 10^−3^	2.952 × 10^−2^
LinkNet++ [29]	ADFECGDB	7.979 × 10^−8^	1.958 × 10^−4^
AEDL [15]	ADFECGDB	3.175 × 10^−6^	2.993 × 10^−2^

**Table 5:** Performance of FQRS detection on the ADFECGDB database by the proposed method.

Data	SE (%)	PPV (%)	F1 (%)
R01	99.46	99.34	99.40
R04	99.14	99.22	99.18
R07	99.30	99.18	99.24
R08	99.23	98.94	99.08
R10	99.07	98.85	98.96
Total	99.24	99.11	99.17

**Table 6:** Comparisons of different methods for FQRS detection on the FECGSYNDB, ADFECGDB, and PCDB datasets.

Method	Database	Se (%)	PPV (%)	F1 (%)
RCED-Net [26]	ADFECGDB	96.06	92.25	94.10
	PCDB	92.60	94.68	93.62
CycleGAN [28]	FECGSYNDB	95.90	96.30	96.10
	ADFECGDB	99.40	99.60	99.70
	PCDB	96.80	97.20	97.90
1D-CycleGAN [17]	ADFECGDB	97.69	94.87	96.26
AEDL [15]	PCDB	97.36	98.68	98.02
	NIFECGDB	91.21	94.65	92.87
DPSS [14]	ADFECGDB	97.30	98.09	97.70
	PCDB	94.20	96.50	95.30
W-Net [18]	ADFECGDB	98.23	99.30	98.81
	PCDB	95.74	97.45	96.91
W-NETR [30]	ADFECGDB	99.45	99.50	99.88
	PCDB	97.82	98.60	98.21
The proposed Attention R2W-Net	FECGSYNDB	97.43	97.98	97.56
	ADFECGDB	99.24	99.11	99.17
	PCDB	98.23	97.74	98.03
	NIFECGDB	96.76	97.39	97.08

**Table 7:** Performance comparison of different convolution modules in FECG signal extraction.

	SE	PPV	F1
Forward convolution block	96.12	96.34	96.41
Residual convolution block	98.52	97.96	98.29
Recurrent convolution block	98.74	98.20	98.50
RRC block	99.24	99.11	99.17

## Data Availability

The data used to support the findings of this study are included in the article.
